# Machine learning assisted analysis of rice flower opening times using a low-cost time-lapse camera

**DOI:** 10.1007/s10265-025-01650-8

**Published:** 2025-06-13

**Authors:** Tomoaki Muranaka, Moeka Matsuura, Kan Yokoyama, Yuuki Gatayama, Satoru Taura, Katsuyuki Ichitani, Eiji Kanda

**Affiliations:** 1 Graduate School of Bioagricultural Sciences, Nagoya University, Furo-Cho, Chikusa, Nagoya, 464-8601 Japan; 2 Faculty of Agriculture, Kagoshima University, 1-21-24 Korimoto, Kagoshima, 890-0065 Japan; 3 Graduate School of Agriculture, Forestry and Fisheries, Kagoshima University, 1-21-24 Korimoto, Kagoshima, 890-0065 Japan; 4 Institute of Gene Research, Kagoshima University, 1-21-24 Korimoto, Kagoshima, 890-0065 Japan

**Keywords:** Environmental response, Flower opening time, Machine learning, *Oryza sativa*, Time-lapse imaging

## Abstract

Flower opening time (FOT) is a key trait for successful reproduction and reproductive isolation. In crop science, FOT is critical for stress avoidance and efficient breeding practices. This study developed a system for the automatic detection of rice flower openings and FOT estimation by integrating a low-cost time-lapse camera with machine learning technology. This approach enabled high-resolution monitoring of flowering dynamics in two cultivars: the *japonica* cultivar Taichung 65 (T65) and the *indica* cultivar IR24. The system accurately identified regions containing open flowers, and the estimated FOTs varied within a 3-h range, with a root mean square error of approximately 30 min compared to manual detection. A significant difference in estimated FOTs between IR24 and T65 demonstrated the system's potential for genetic screening applications. FOT of both cultivars exhibited a significant negative correlation with daily mean temperature. Notably, a temperature-sensitive period was identified in the morning, suggesting that temperature influences not only flower opening but also preceding physiological processes such as panicle and spikelet development. This study presents a novel approach to investigating FOT dynamics in rice and provides insights into the interaction between environmental factors and internal regulatory mechanisms governing this critical reproductive trait.

**Supplementary Information:**

The online version contains supplementary material available at 10.1007/s10265-025-01650-8.

## Introduction

The regulation of flowering time is critical for successful reproduction and reproductive isolation (Franks 2015; Matsumoto et al. 2013; Stam 1983). In addition to the flowering season, the flower opening time (FOT) within a day is also important (Van Doom 2003; Van Doom and Kamdee 2014). Temporal synchrony between flower opening and insect activity is essential for efficient pollination (Bloch et al. 2017; Fenske et al. 2018). Differences in FOT can serve as an isolating barrier among closely related species in sympatric habitats (Liu et al. 2020; Subba Reddi et al. 1988). In various plant species, it has been established that the circadian clock, an internal timing mechanism based on diel rhythms, plays the primary role in regulating FOT by setting a "timing gate" for flower opening (Liu et al. 2022; Marshall et al. 2023; Muroya et al. 2021; Overland 1960; Yon et al. 2016) . As a secondary layer of regulation, flowers respond to environmental cues to adjust their FOT (Nishiyama and Blanco 1981; Van Doom 2003).

FOT is a key phenotype for crop performance. High-temperature stress during the reproductive stage causes significant damage in cereals (Jagadish 2020; Prasad et al. 2017). Earlier FOT can help crops escape heat stress during the afternoon (Bheemanahalli et al. 2017). In rice (*Oryza sativa* L.), there are two major subspecies: *indica* and *japonica*. The *indica* cultivars are predominantly grown in tropical areas, while the *japonica* cultivars are mainly cultivated in temperate regions. Typically, *indica* cultivars have earlier FOTs than *japonica* cultivars due to local adaptations (Che et al. 2024; Wang et al. 2023). With the ongoing effects of global warming, high-temperature damage to rice grains has been reported even in temperate regions (Hasegawa et al. 2011; Liu et al. 2023; Yoshimoto et al. 2011). As a result, FOT has become an increasingly important trait in breeding. On the other hand, FOT differences can present challenges in hybrid breeding, because it is difficult to cross *indica* and *japonica* under natural conditions due to their FOT differences despite their potentially higher yield compared to intra-subspecies hybrids (Li and Ouyang 2022; Qian et al. 2016).

Given these factors, genetic analysis of FOT has gained momentum in recent years. FOT-related quantitative trait loci (QTL) have been identified using an FOT mutant in the *japonica* cultivar Nipponbare (Zhang et al. 2017). The incorporation of a QTL (*qEMF3*) from the wild rice species *O. officinalis* advanced FOT by approximately 1.5 to 2.0 h in the *indica* cultivars Nanjing 11 and IR64 (Hirabayashi et al. 2015). Genes that regulate pectin methyl-esterification levels in the lodicule cell walls also influence FOT, as increased pectin methyl-esterification leads to cell wall softening, facilitating water absorption by lodicules and promoting flower opening (Wang et al. 2022). The introgression of the *indica* haplotype of *OsMYB8*, which induces jasmonic acid (JA) isoleucine synthetase, has been shown to effectively promote FOT in *japonica* cultivars (Gou et al. 2024). Similarly, overexpressing *OsMYC2* in *japonica* increased JA content and decreased cellulose and hemicellulose contents in lodicule cells, leading to 1.5 h earlier FOT (Zhu et al. 2024). Furthermore, the genetic manipulation of other JA regulators sufficiently altered FOTs (Wang et al. 2024). These recent findings highlight the importance of JA as an accelerator for the flower opening in rice.

Beyond genetic variation, environmental factors affecting FOT have also been studied in rice. Higher temperatures, radiation, and humidity in the morning lead to earlier FOT, with the relative contributions of these factors varying across cultivars (Kobayasi et al. 2010). Extended darkness delayed flower opening in IR64, resulting in a significant advancement of FOT the following day (Ishimaru et al. 2021). Despite the accumulation of knowledge, the full mechanisms underlying FOT determination remain unclear, as flower opening involves multiple steps, including developmental, physiological, and physical processes. Related to this, there are many unknowns regarding which processes are affected by the environment, and which times of day are more affected. Additionally, the flowering process at the organ and whole plant levels is highly complex. In rice, flowering occurs over several days at the whole plant level, yet each flower opens only once and closes within an hour or less. A single rice plant in a paddy field typically produces 10–20 panicles, each bearing 50–200 spikelets. These spikelets open sequentially in coordination with panicle development, indicating that rice flowering is temporally and spatially regulated.

To investigate the mechanisms underlying the spatiotemporal regulation of flowering, crops serve as an ideal model, as they enable the production of large numbers of genetically homogeneous plants with abundant flowers. However, due to this genetic homogeneity, the flowering period is limited, making it challenging to collect sufficient data. To overcome this limitation, we employed a weekly sowing strategy in rice, a technique commonly used in breeding programs to ensure a continuous supply of pre-heading plants throughout the reproductive season.

Time-lapse imaging with high temporal resolution is a powerful method for capturing the spatiotemporal dynamics of flower openings. Recently, compact cameras designed for long-term field monitoring have become widely available at low cost, primarily for wildlife observation and security applications. We utilized such a camera to record the dynamics of the rice flower opening. To efficiently analyze a large volume of images, we developed an automated system that determines the FOT from time-series images using YOLOX, an object detection algorithm based on machine learning. The accumulation of spatiotemporal flowering dynamics under diverse environmental conditions will provide valuable insights into how genetic factors link to the developmental and environmental regulations of FOT.

## Materials and methods

### Plant material

A *japonica* rice cultivar, Taichung 65 (T65), and an *indica* rice cultivar, IR24, were used in this study. These two cultivars were sown weekly to ensure pre-heading plants throughout the experimental periods (Table S1). Germinated seeds were initially sown in nursery beds within a greenhouse in the Experimental field of the Faculty of Agriculture, Kagoshima University, Kagoshima, Japan (31°34′23.3"N, 130°32′35.8"E, 7 m). Following Ichitani et al. (2014), the seedlings were transplanted in a paddy field near the greenhouse. The five cohorts of seedlings sown earlier were transplanted in pots (diameter: 18 cm, height: 20 cm, 4 L) filled with field soil containing 3 g of NPK 15-15-15 fertilizers placed in the experimental field approximately four weeks after sowing date, because the paddy fields were available only between mid-June and November. Approximately two months after transplanting, the plants started heading. For each cultivar, more than 12 plants were prepared for each cohort and two plants at a pre-heading or early heading stage were selected. Plants grown in the paddy field were transferred to pots the day before they were used for imaging.

### Time-lapse recording

**Fig. 1:**
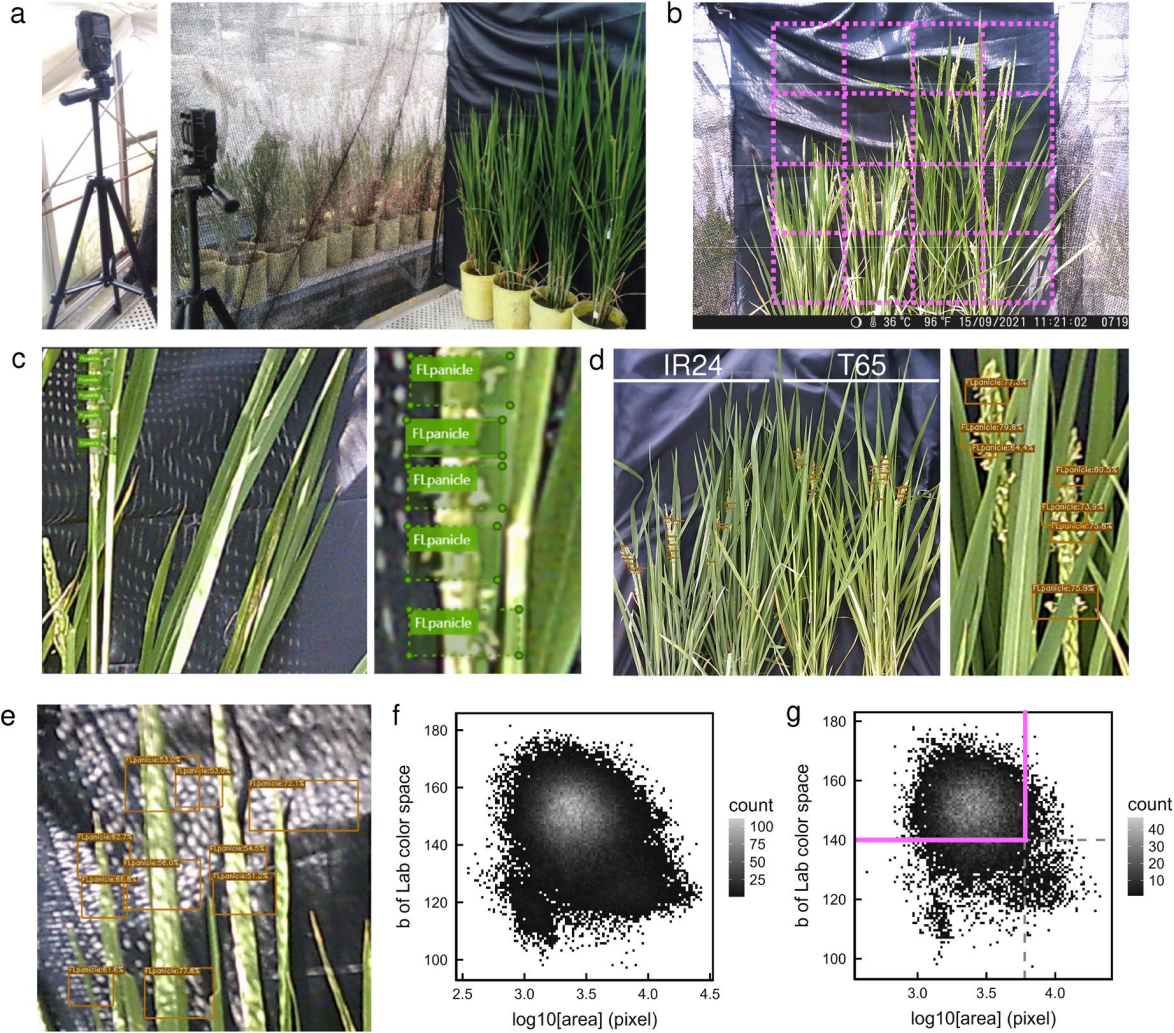
Overview of flower-opening detection by YOLOX. **a** A time-lapse camera with a tripod (left) was set on a table in the glasshouse to capture side-view images of four pots (right). **b** The 16 regions of 700 × 700 pixels used as input for YOLOX. **c** The annotated image (left) and a close-up view (right). **d** The entire image after flower-opening detection for two IR24 pots and two T65 pots (left), and a close-up view of flowering panicles of T65 (right). **e** Example of false detections. **f** Density plot of the mean *b* value and area of detected regions with a score higher than 0.5. **g** Density plot of the mean *b* value and area of detected regions with a score higher than 0.7. Magenta lines indicate thresholds for false detections

Two plants from each cultivar were placed side by side on a potting table in the greenhouse, and images were captured every 10 min using the time-lapse mode of a trail camera (DVR-Z4, Hanwha Q CELLS Japan) set on a tripod at 120 cm from plants (Fig. 1a). While the trail camera is relatively compact (104 mm × 75 mm × 43 mm) and affordable (5900 yen) compared to other models, it includes all the essential features for long-term field monitoring, such as waterproof and dustproof protection (IP66), night vision imaging with built-in infrared light, and an extended operating time of up to two weeks with 10-min interval imaging using four rechargeable AA batteries.

The image resolution was set to 4608 × 3456 pixels. To prevent halation, the plants were covered with 50% blackout nets, and a black cloth was positioned behind them as a backdrop to avoid backlighting. Temperature, humidity, and radiation were recorded every 10 min using a data logger equipped with sensors (TR-74Ui, T&D Corporation). When most of the flowers on the monitored plants had opened, the plants were replaced with pre-heading plants. Time-lapse recordings of approximately 10 days were conducted four times from 9/9 to 10/19 in 2021, and six times from 7/30 to 10/5 in 2022 (Table S2). Note that one T65 and three IR24 plants were monitored from 9/3 to 9/14 in 2022 because there was no other suitable T65 plant.

### FOT estimation using YOLOX

Machine learning-based object detection was identified as a suitable method for detecting open flowers in the collected images. Due to its high performance and ease of installation, YOLO (You Only Look Once) has become a popular tool for object detection (Redmon et al. 2016). In this study, we used YOLOX, a more efficient version with a simplified design (Ge et al. 2021). YOLOX was cloned from GitHub (https://github.com/Megvii-BaseDetection/YOLOX) and run in a Pytorch and Anaconda environment. The Python scripts, training dataset, and trained model used in this analysis are available on GitHub (https://github.com/mwbotan/FLpanicle).

To train the model, we selected 24 images from the 2021 dataset, each containing a large number of open flowers. The original 2800 × 2800-pixel regions, encompassing four plants, were divided into 16 smaller images of 700 × 700 pixels (Fig. 1b). These images were further resized to 640 × 640 pixels before being fed into YOLOX. Open-flower regions in the images were annotated using VoTT (Visual Object Tagging Tool, Microsoft) with the “FLpanicle” tag (Fig. 1c). Since individual flowers were too small to annotate separately, well-flowered regions on the panicles were marked using the smallest possible rectangular bounding boxes. Both cultivars were annotated collectively without distinction. In total, 441 annotations were made across 189 images of 700 × 700 pixels. The annotated dataset was then converted to COCO format using an open-source Python script (https://github.com/Kazuhito00/convert_voc_to_coco).

For model training, the 151 images containing 368 annotations were used as the training dataset, while the remaining 38 images with 73 annotations were used as validation dataset. The training process employed the *yolox_s* model with the options -b 4 –fp 16. After 72 training iterations, the best-performing model was selected based on its highest evaluation metrics (average precision [AP] = 12.818, average recall [AR] = 30.411). The relatively low AP score may be attributed to the region-level annotation, which made it difficult to precisely match the predicted boxes and validation boxes. The trained model was converted to the Open Neural Network Exchange (ONNX) format using a built-in YOLOX script.

The trained model was applied to detect open flowers in images collected in 2022. Each image was first divided into 16 sections of 700 × 700 pixels, and YOLOX was used to identify regions containing open flowers. These detected regions were then reassembled into a composite image of 2800 × 2800 pixels by integrating the 16 sections. The model successfully detected flowering regions in both IR24 and T65 cultivars (Fig. 1d). When the confidence threshold was set to 0.5, some false positives were observed, primarily due to shadows from blackout nets used to prevent glare in the images (Fig. 1e).

To filter out false positives, additional criteria were applied based on confidence score, area size, and color characteristics. Since correctly detected flowering regions were expected to overlap with mature panicles, which appear yellow, color filtering was employed using the *b* value in the Lab color space. Image color spaces were converted from RGB to Lab using OpenCV functions, and the mean *b* value was calculated for each detected region. A scatter plot of *b* values versus detected area sizes revealed a distinct cluster centered around a *b* value of 150 and an area size of 2500 pixels (~ 3.4 in log10 scale), which was considered to correspond to correctly identified flowering regions (Fig. 1f). Based on these observations, the filtering criteria were manually adjusted to reduce false positives while retaining correctly detected flowers. The final thresholds were set as follows: confidence score > 0.7, mean *b* value > 140, and area < 6000 pixels (Fig. 1g).

To quantify flower opening dynamics, a flower index was calculated as the sum of the heights of the filtered regions, based on the assumption that region height correlates with the number of open flowers. The left half (x < 1350 pixels) and right half (x > 1450 pixels) of each image were analyzed separately to correspond to each cultivar. For images collected from September 3 to September 14, the regions on the left (x position < 650 pixels) and the right (x position > 750 pixels) were analyzed separately due to the different plant layouts (Table S2). The resulting time series of flower indices were fitted using smooth spline function built in R 4.3.3, and the time for the highest peak time was determined as the FOT (Fig. S1). For the validation of the estimated FOT, the image where the number of opening flowers was likely highest in a day was visually searched for each cultivar. The recorded time of the image was determined as the observed FOT.

### Calculating humidity deficit

The humidity deficit (HD) was calculated using the following equation, where T is temperature and H is humidity (Junzeng et al 2012).

$$HD= \frac{217 }{T+273.15} \times (6.11 \times 10^( \frac{7.5 \times T}{T+237.3} ))\times (\frac{100-H}{100})$$

### Statistical analysis

Statistical tests in this study were performed using R 4.3.3 with its built-in functions. For correlation, *cor.test* function was used with default settings. For boxplots, *pairwise.wilcox.test* function was used with default settings.

## Results

### The open flowers of both rice cultivars were detectable using YOLOX

To compare the flower-opening times between the two cultivars, the *japonica* rice cultivar T65 and the *indica* rice cultivar IR24 were used in this study. Side-view images of two plants from each cultivar, grown in pots, were captured every 10 min using a time-lapse camera (Fig. 1a). Time-lapse recordings were conducted in September and October 2021, and again in August and September 2022. The plants were replaced with new pre-heading plants approximately every 10 days. We trained the YOLOX model with annotations of open flowers for images obtained in 2021 and then detected open flowers in the 9,325 images obtained in 2022 using the trained model with the filter to reduce false positives (Fig. 1c-g). See the “Material and methods” section for more details.

**Fig. 2:**
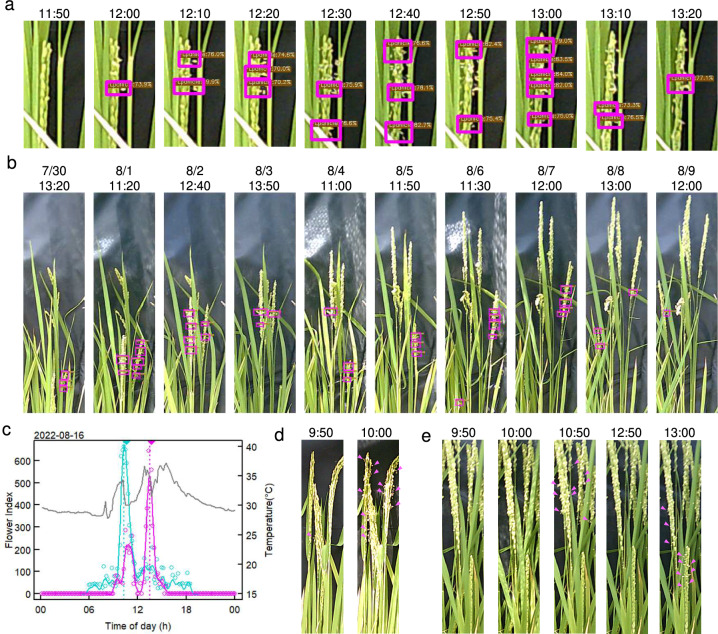
Estimation of FOT. **a** Example of flower opening detection by YOLOX. T65 panicle on 2022-8-13. Magenta rectangles represent the detected region. **b** Daily changes of the flower opening region in T65 panicles. Magenta rectangles represent the detected region. **c** Diel changes in the flower index on 2022-8-16. Cyan and magenta represent IR24 and T65, respectively. Colored lines are smooth splines. Dotted lines and closed triangles represent estimated and observed FOT, respectively. Air temperature is shown as a black line. **d**, **e** Examples of flowering panicles in IR24 (**d**) and T65 (**e**) on 2022-8-16. Magenta arrows indicate open flowers

The YOLOX-based detection of open flowers successfully captured the spatiotemporal dynamics of flower opening. Flowers with visible stamens protruding outward were recognized as open, whereas flowers with withered stamens pointing downward were not detected as open (Fig. 2a). The detection accuracy for flower closure was sufficiently high to reliably capture the brief flower-opening state, which lasts for approximately 30 min.

The flower opening continued for approximately 5 days at a panicle level and for more than 10 days at a whole plant level (Fig. 2b). Initially, the flower opening was concentrated in the upper region of the panicle. As panicle development progressed, the region of active flower opening gradually shifted downward, reflecting the maturation sequence of spikelets. Since the detected regions often contained multiple open flowers, the total height of detected regions was considered a more reliable index for flower opening than the absolute number of detected regions.

To estimate FOT, the flower index for each cultivar was calculated for all images. In most cases, a single sharp peak was observed for each cultivar (Fig. S1). However, additional peaks were occasionally detected. For example, on 2022-8-16, IR24 exhibited a single peak, while T65 showed two peaks (Fig. 2c). We confirmed that the peak in IR24 corresponded to the synchronous flowering of individual panicles within a 10-min interval (Fig. 2d). For T65, two distinct peaks of flower-opening were observed on the same day (Fig. 2e). The earlier peak corresponded to the flower openings on the middle parts of mature panicles. In contrast, the later peak corresponded to flower openings on the lower parts of mature panicles and a younger panicle, indicating a relationship between the FOT and the developmental stages of the panicles. Notably, the earlier peak of T65 overlapped with the peak of IR24, although the exact peak times differed by 50 min (Fig. 2c-e). This implies that a common environmental signal may trigger flower opening in both cultivars.

Although peak splitting was observed only once in T65, IR24 exhibited multiple instances of peak splitting on different days (Fig. S2). In some cases, the sequence of flowering aligned with the developmental stage, with the earlier peak occurring in the mature part of the panicle (Fig. S2b, c). However, in other cases, two or three distinct flower-opening events were observed in the same region of a panicle within a day, suggesting heterogeneous flower states within the panicle (Fig. S2a, d).

### FOTs varied across days and showed significant differences between cultivars

**Fig. 3:**
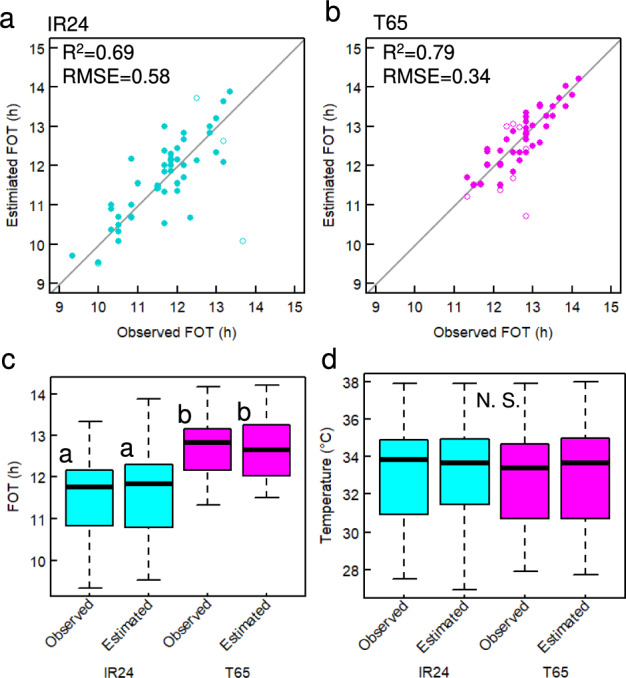
Estimated and observed FOT of IR24 and T65. **a**, **b** Comparison of estimated and observed FOT for IR24 (**a**) and T65 (**b**). Days on which fewer than three detected regions were recorded are shown as open points and were excluded from further analysis. Black lines represent estimated = observed. **c** Boxplots of estimated and observed FOT for IR24 and T65. Different letters indicate significant differences based on the pairwise Wilcoxon test (*p* < 0.05). **d** Boxplots of air temperature at each FOT. No significant differences were found based on the pairwise Wilcoxon test

The FOT for each day was defined as the time when the smoothed flowering index reached its peak. To validate the estimated FOT, we manually identified the observed FOT for each day by visually searching for the image with the most flowers opening. Observed FOTs were manually determined for 48 days for IR24 and 50 days for T65. The observed FOT for IR24 ranged from 9:20 to 13:40, while for T65, it ranged from 11:20 to 14:10. The estimated FOTs captured inter-day variation with a root mean square error (RMSE) of 0.58 h (~ 34.8 min) for IR24 and 0.35 h (~ 21 min) for T65 (Fig. 3a, b). These low RMSEs suggest that the proposed FOT estimation method is highly practical. Note that data from four days for IR24 and eight days for T65 were excluded due to a small number (< 3) of detected flowers.

The mean estimated FOT for IR24 (11:41 ± 61 min, mean ± SD) matched that of the observed FOT (11:41 ± 57 min, mean ± SD). Similarly, the mean estimated FOT for T65 (12:41 ± 43 min, mean ± SD) matched that of the observed FOT (12:41 ± 44 min, mean ± SD). The FOT of IR24 was significantly earlier than that of T65 (Fig. 3c). No significant differences were observed between the estimated and observed FOTs in either cultivar, further confirming the high practicality of the FOT estimation method for cultivar comparison. We also compared the temperature at the FOT between IR24 and T65, because earlier FOTs are expected to avoid high temperatures (Fig. 3d). However, no significant differences were found between the cultivars, likely due to the relatively small difference in FOT.

### FOT showed a significant negative correlation with temperature

**Fig. 4:**
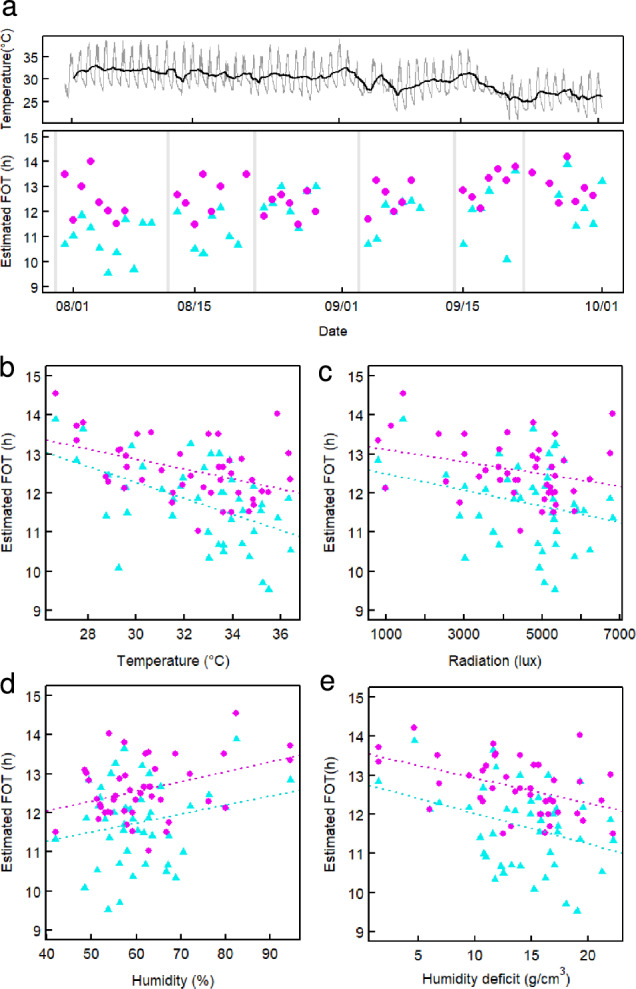
Relationship between FOT and weather factors. **a** Temporal changes in air temperature (top) and estimated FOT (bottom). Gray and black lines indicate 10-min interval raw data and average temperature for the past 24 h, respectively. Cyan triangles and magenta circles represent IR24 and T65, respectively. Gray bars indicate the days when the monitored plants were renewed. **b**, **e** The estimated FOT of IR24 (cyan triangle) and T65 (magenta circle) plotted against temperature (**b**), radiation (**c**), humidity (**d**), and humidity deficit (**e**). Each weather factor represents the mean value from 8:00 to 15:00. Dotted lines represent linear regressions

We then investigated factors contributing to inter-day variation in FOT. In IR24, FOT showed a significant correlation with the date (*R* = 0.50, *p* = 0.00058), whereas in T65, no significant correlation was observed (*R* = 0.30, *p* = 0.055) (Fig. 4a). In both cultivars, FOT difference between neighboring days was substantial, with an average difference of 54 ± 46 min in IR24 and 52 ± 33 min in T65 (mean ± SD). This suggests that FOT is influenced more by fluctuating weather conditions than by differences in day length which gradually changes across seasons.

To further explore this, we examined the relationship between FOT and three recorded weather factors: temperature, radiation, and humidity. FOT for each day was plotted against the daily mean values (from 8:00 to 15:00) of these factors (Fig. 4b–d). Temperature was the only factor that showed a significant negative correlation with FOT in both IR24 and T65 (Table 1). In contrast, radiation and humidity significantly influenced FOT in T65 but not in IR24. Higher radiation and lower humidity led to earlier flowering in T65, the similar trend also observed in IR24. It is important to note that temperature was significantly correlated with both radiation (*R* = 0.67, *p* = 7.0e−7) and humidity (*R* = −0.56, *p* = 8.5e−5), suggesting that temperature may partially account for the observed correlations with these factors. Additionally, we estimated the humidity deficit based on temperature and humidity (Fig. 4e). While the humidity deficit showed significant negative correlations with FOT in both cultivars, these correlations were weaker than those observed with temperature.

**Table 1:** Correlation between FOTs of the two cultivars and weather factors

Factor	IR24		T65	
	*R*	*P* value	*R*	*P* value
Temperature	−0.50	0.00058	−0.48	0.0014
Radiation	−0.27	0.075	−0.33	0.031
Humidity	0.22	0.15	0.39	0.010
Humidity deficit	−0.34	0.026	−0.45	0.0030

Overall, these results indicate that temperature is the primary determinant of FOT. The relatively high sensitivity of IR24 FOT to air temperature may explain why its FOT was delayed in the later part of the monitoring period as temperatures declined.

### FOT was more sensitive to the temperature during specific times of the day.

**Fig. 5:**
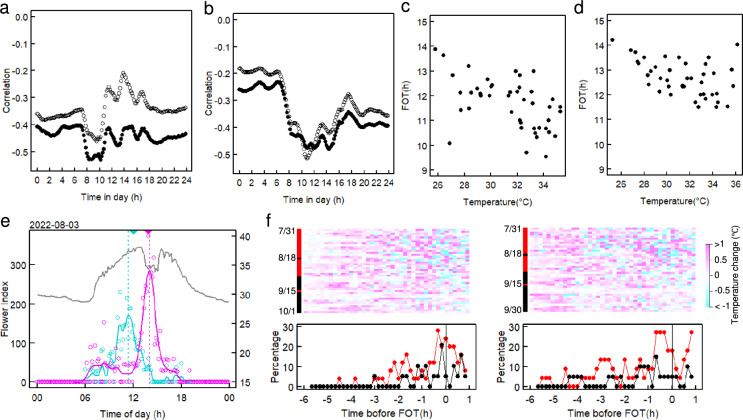
Relationship between FOT and temperature. **a,** **b** Time series of correlation coefficients between FOT and the average temperature for the past 1 h at each time point for IR24 (**a**) and T65 (**b**). Closed and open circles represent estimated and observed FOT, respectively. **c** The estimated FOT of IR24 plotted against the average temperature between 9:10 and 10:10. **d** The estimated FOT of T65 plotted against the average temperature between 9:50 and 10:50. **e** Diel changes in the flower index on 2022-8-3. Cyan and magenta represent IR24 and T65, respectively. Colored lines are smooth splines. Dotted lines and closed triangles represent estimated and observed FOT, respectively. Air temperature is shown as a black line. **f** Relationship between temperature fluctuations and FOT for IR24 (left) and T65 (right). The heatmaps show temperature differences over 10-min intervals. Red bars and black bars on the left side represent days when the maximum temperature was above or below 35 °C, respectively. The line graphs show the percentage of days on which temperature decreases larger than 0.5 °C occurred within 10 min for days when the maximum temperature was above 35 °C (red) or below 35 °C (black) at each time point

The flower-opening process in rice involves a sequence of physiological, developmental, and physical events (Wang et al. 2023). To investigate whether temperature affects a specific phase of this process, we calculated the correlation between FOT and the average temperature during the previous 1-h for each time point. In IR24, the strongest negative correlation was observed at 10:10 (*R* = −0.53, *p* = 0.00021) for estimated FOTs and at 9:30 (*R* = −0.46, *p* = 0.0016) for observed FOTs (Fig. 5a). For T65, the strongest negative correlation was observed at 14:20 (*R* = −0.48, *p* = 0.0013) for estimated FOTs and at 10:50 (*R* = −0.52, *p* = 0.00048) for observed FOTs. The negative correlation for estimated FOTs at 10:50 (*R* = −0.48, *p* = 0.0015) was equivalent to the correlation at 14:20. Both cultivars exhibited approximately 2-h temperature-sensitive phases, with IR24's phase occurring earlier than T65’s, consistent with the observed FOT order.

To explore additional factors influencing FOT, we plotted FOT against the average temperature of the previous 1-h at the time point exhibiting a strong correlation during the temperature-sensitive phases (Fig. 5c, d). While higher temperatures generally led to earlier flowering, extremely high temperatures delayed flowering, particularly in T65. In some instances, the flower index peak in T65 coincided with a temporary drop in temperature (Fig. 5e, other examples in Fig. S1: “2022-8-6”, “2022-8-13”, “2022-8-15”, 2022-8-16”, “2022-8-18”).

We then examined the relationship between FOT and temperature fluctuations (Fig. 5f). In both IR24 and T65, around 30% of the hot days, those with a maximum temperature exceeding 35 °C, showed a temporary temperature decrease of more than 0.5 °C within 10 min prior to FOT, while such temperature drops were observed on only about 10% of non-hot days except the single time point of IR24, 10-min before FOT. These results suggest that warmer temperatures accelerate FOT, but extremely high temperatures may delay it, with temporary temperature decreases potentially acting as a trigger for flower openings.

## Discussion

Over the past decade, rapid advancements in information technology have led to smaller and more affordable devices. The application of these technologies in biological research is expected to accelerate scientific progress. This study demonstrated that combining a low-cost time-lapse camera with machine learning tools can effectively capture rice flowering dynamics at high temporal resolution with minimal effort. While the precision of flower detection may not significantly surpass that of previous studies, our system is considerably easier to use, as it integrates a commercially available time-lapse camera with open-source software packages employing single-shot object detection algorithms (Guo et al. 2015). Additionally, the number of images required for training was only about one-tenth of that needed in previous research using neural networks (Desai et al. 2019).

Although the estimated FOTs for both the *japonica* cultivar T65 and the *indica* cultivar IR24 varied within a three-hour range, IR24 exhibited a significantly earlier FOT than T65 in our system. This suggests that the developed system can effectively detect cultivar-specific differences and is practical for genetic screening. However, the precision of FOT estimation was lower for IR24 than for T65, possibly due to differences in panicle and stamen morphology. Expanding the training data to include additional cultivars may improve estimation accuracy across diverse genetic backgrounds.

Compared to the side-view imaging used in this study, top-view imaging may be more suitable for large-scale screening or field applications. Further validation is needed to determine whether the developed system can achieve comparable performance to previous studies using top-view imaging (Desai et al. 2019; Guo et al. 2015). Additionally, the impact of environmental factors such as rain and wind on the accuracy of flowering detection should be further investigated for field applications.

In this study, flowering dynamics were observed over approximately 40 days. FOT exhibited a negative correlation with daily mean temperature, consistent with previous studies (Kobayasi et al. 2010). In contrast, no strong correlation was found with radiation or humidity, likely due to the controlled conditions inside the glass greenhouse. Although the humidity deficit, calculated from temperature and humidity, showed a significant negative correlation with FOT, this may be attributed to its strong correlation with temperature.

The responses to weather factors were similar between T65 and IR24, suggesting that the effects of temperature and radiation on FOT are somewhat conserved across subspecies. However, IR24's FOT was more influenced by temperature, whereas T65's FOT was significantly affected by radiation and humidity. Overall, temperature appears to be the primary factor regulating FOT. Interestingly, FOT was more sensitive to temperature in the morning, with a two-hour window of influence. The sensitive periods differed between T65 and IR24, aligning with their respective FOT timings. This suggests that not only the timing of FOT but also the preceding flower development processes, including temperature-sensitive reactions such as enzyme activity, are regulated in different phases of the circadian clock between the two cultivars (Wang et al. 2020).

The side-view imaging revealed the spatial heterogeneity of flower openings. On some days, splitting peaks of flower openings were observed within the same individual. Panicles that had flowered the previous day bloomed earlier, while elongating panicles flowered later, implying that FOT regulation differs between elongating and fully elongated panicles. This may be due to the same mechanism that accelerates flowering the next day when the flowering was inhibited by extended darkness (Ishimaru et al. 2021).

The importance of JA in the flower opening process has been established (Wang et al. 2024; Zhu et al. 2024). Methyl JA (MeJA) treatment promotes panicle flowering but increases sterility because open flowers include immature flowers which had been expected to open the next day of the treatment day (Kobayashi and Atsuta 2010). Thus, flower opening is strictly coupled with its maturation. The splitting flowering peaks in the same panicle may represent the heterogeneity of maturations.

The splitting peaks were observed at the early period of monitoring, from July 31 to August 1, when the air temperature was high (Figs. S1, S2). Note that the earlier peak of splitting peaks of IR24 flowering on 2022-9-10 (see Fig. S1) was due to false positives. Rapid growth under high-temperature conditions might have contributed to heterogeneous maturation within panicles. We should also note that the plants used in the early period were precultured in a pot, therefore the limitation of the nutrient uptake with small rhizosphere also might cause heterogeneous maturations (Table S2).

In most cases, flowering was highly synchronized both within and between individual plants. Although the precision of the circadian clock in rice has been reported to be 22 min, cellular pathways are inherently heterogeneous and subject to biological noise (Matsuzaki et al. 2015; Muranaka and Oyama 2016). Interestingly, synchronization was sometimes observed between the two cultivars despite their genetically distinct FOTs, suggesting that external cues, in addition to internal controls, may influence flowering synchronization (Van Doorn 2003; Van Doorn and Kamdee 2014).

Since physical processes such as lodicule water absorption and lemma and palea transpiration play key roles in rice flower opening, it is important to consider the impact of microclimate fluctuations on these processes (Ogawa et al. 2019; Wang et al. 2022). In this study, temporary temperature drops may have triggered flower opening on hot days when the maximum temperature exceeded 35 °C. However, this was not consistently observed, highlighting the need for further investigations, including field data collection and controlled indoor experiments. Additionally, plant tissue temperature does not always match air temperature, so other environmental factors such as radiation and wind velocity should also be considered.

Moving forward, developing an easy and high-throughput system for measuring flowering dynamics will be crucial. This study provides foundational insights for the development of machine learning-powered tools for automated flower detection. Variations in FOT play a critical role in sustaining ecosystem biodiversity by influencing reproductive efficiency and isolation mechanisms. By using rice as a genetically uniform and manageable model plant, this study offers valuable perspectives on the interplay between environmental factors and internal regulatory mechanisms underlying this key reproductive trait.

## Supplementary Information

Below is the link to the electronic supplementary material.

Supplementary file1 (PDF 3696 KB)

## Data Availability

The Python scripts, training dataset, and trained model used in this analysis are available on GitHub (https://github.com/mwbotan/FLpanicle). The other data generated and/or analyzed in the current study are available from the corresponding author upon reasonable request.
