# Supplementary material for: Machine learning assisted analysis of rice flower opening times using a low-cost time-lapse camera
Source: J Plant Res. 2025 Jun 13;138(5):733–43. doi: 10.1007/s10265-025-01650-8 (PMC12441105; doi:10.1007/s10265-025-01650-8)
Supplement: Supplementary file 1 — Supplementary file1 (PDF 3696 KB) [file 10265_2025_1650_MOESM1_ESM.pdf]

Title

Machine learning assisted analysis of rice flower opening times with a low-cost time-lapse camera.

Journal of Plant Research

Author information

Tomoaki Muranaka[1*], Moeka Matsuura[2], Kan Yokoyama[2], Yuuki Gatayama[3], Satoru Taura[4], Katsuyuki Ichitani[2], Eiji Kanda[2]

1. Grad. Sch. of Bioagricultural Science, Nagoya University, Furo-cho, Chikusa, Nagoya, Aichi, 464-8601 Japan
2. Faculty of Agriculture, Kagoshima University, Korimoto 21-24-1, Kagoshima 890-0065, Japan.
3. Graduate school of Agriculture, Forestry and Fisheries, Kagoshima University, 1-21-24 Korimoto, Kagoshima, Kagoshima 890-0065, Japan
4. Institute of Gene Research, Kagoshima University, Korimoto 21-24-1, Kagoshima 890-0065, Japan.

Corresponding author

Tomoaki Muranaka

E-mail: muranaka@agr.nagoya-u.ac.jp

(Fig. S1 Continuing to the next page)

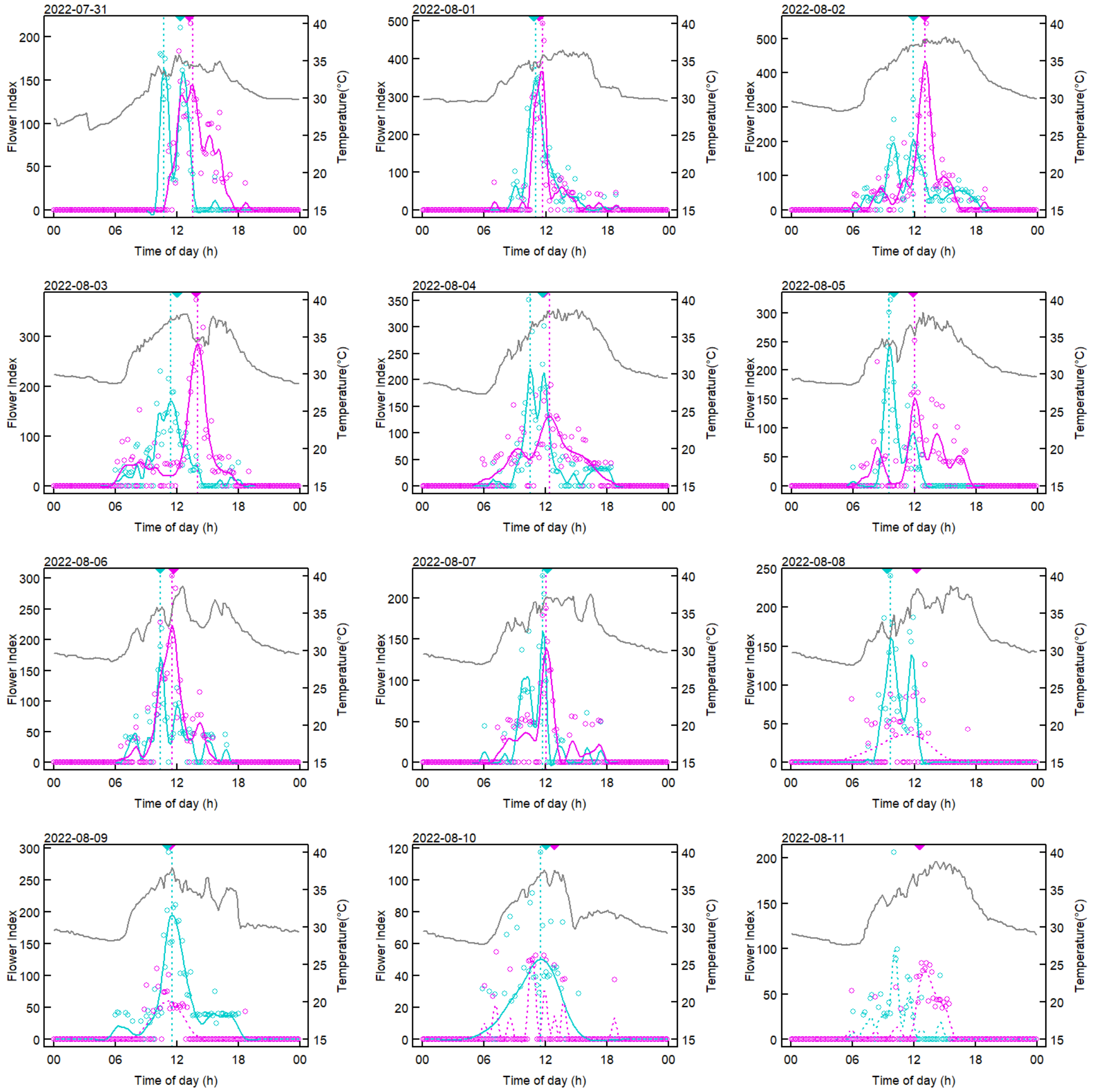

Fig. S1 Diel changes of flower index at all monitored days. Cyan and magenta represent IR24 and T65, respectively. Colored lines are smooth splines. Dotted lines and closed triangles represent estimated and observed FOT, respectively. Dotted lines were not shown when the number of detected regions was less than three flowers. The air temperature was shown as a black line. Each page corresponds to each time-lapse recording with new plants.

(Fig. S1 Continuing to the next page)

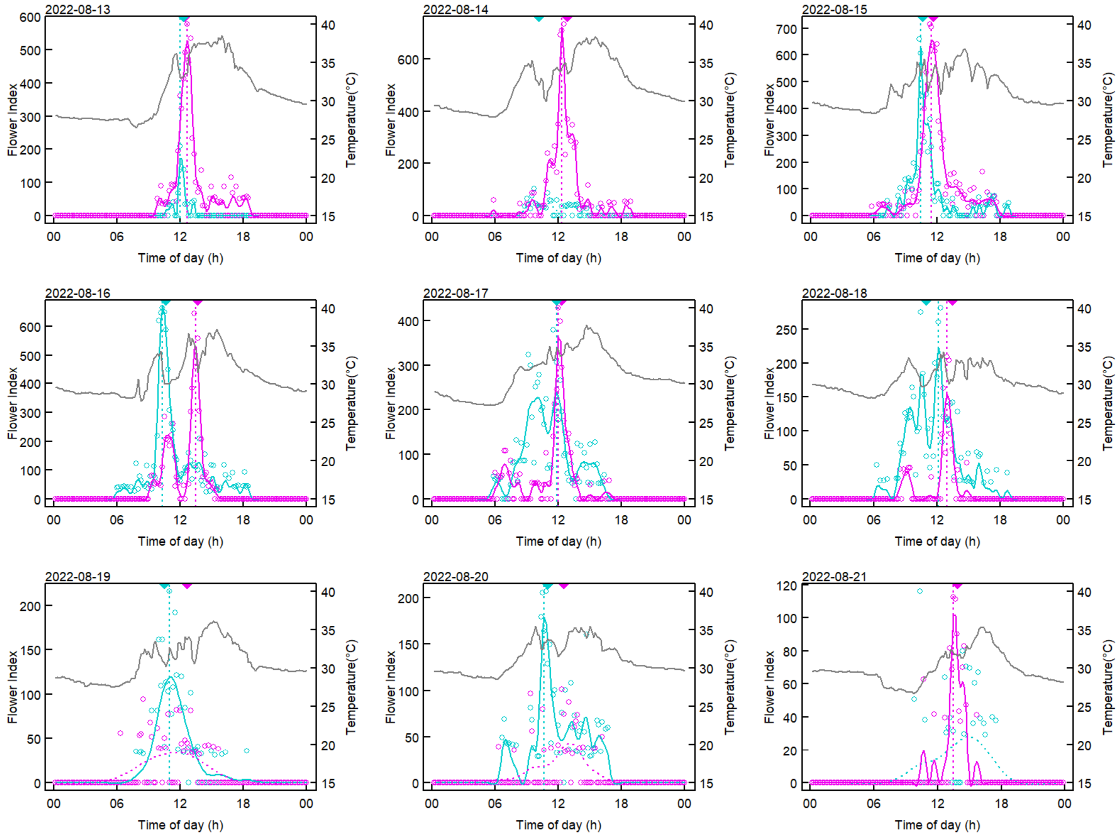

(Fig. S1 Continuing to the next page)

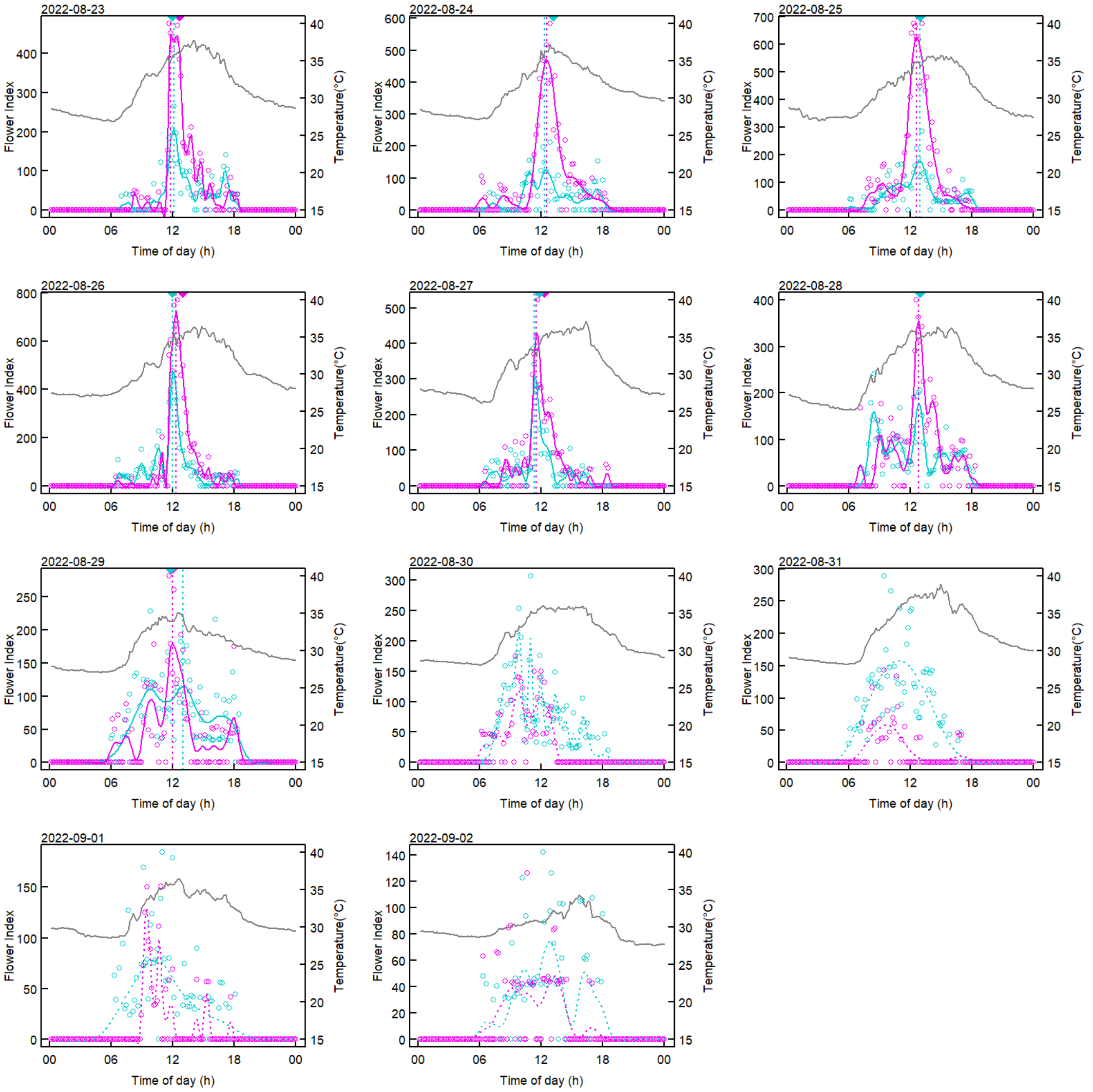

(Fig. S1 Continuing to the next page)

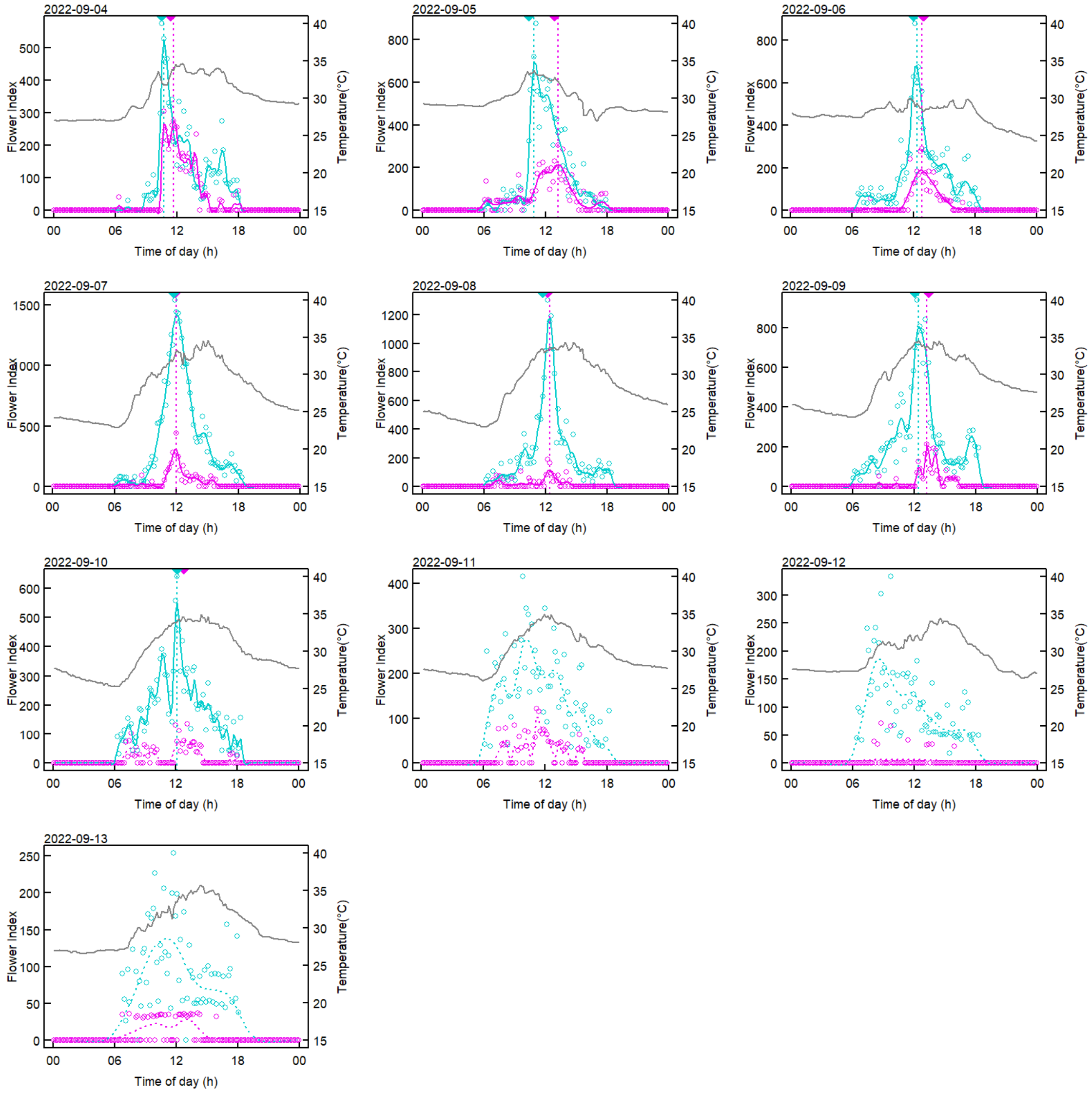

(Fig. S1 Continuing to the next page)

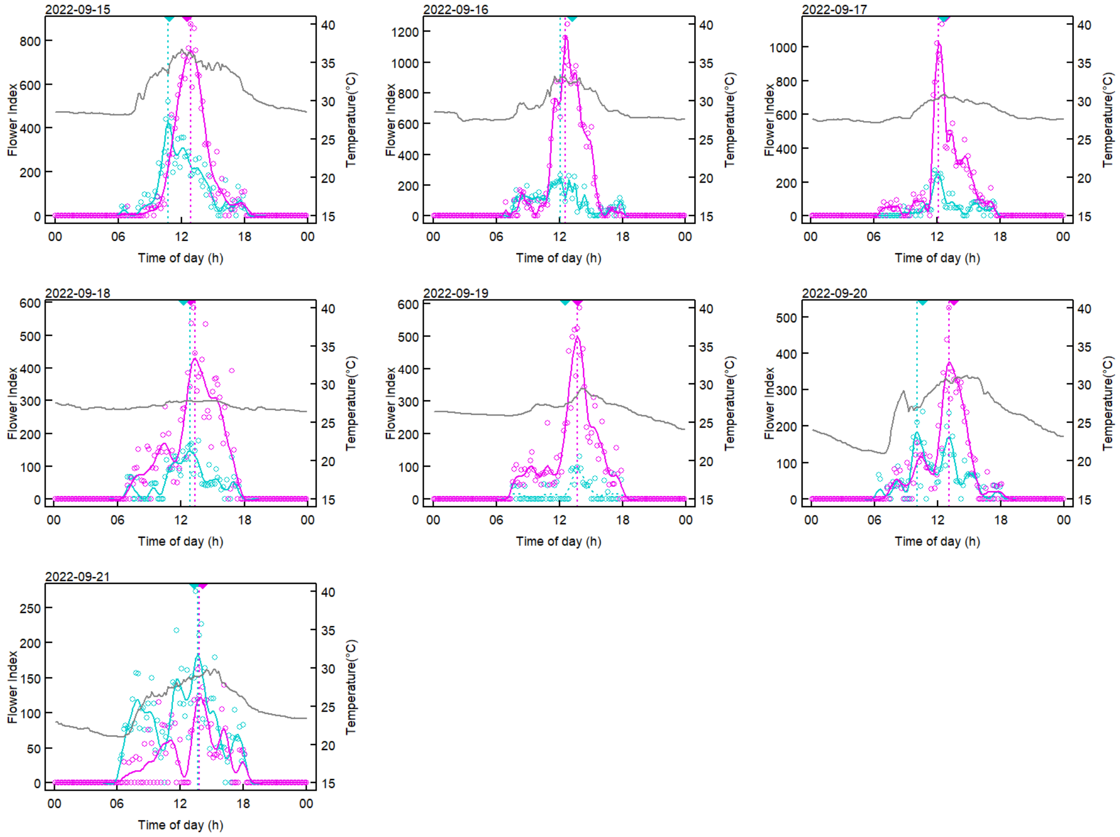

(Fig. S1 Last page)

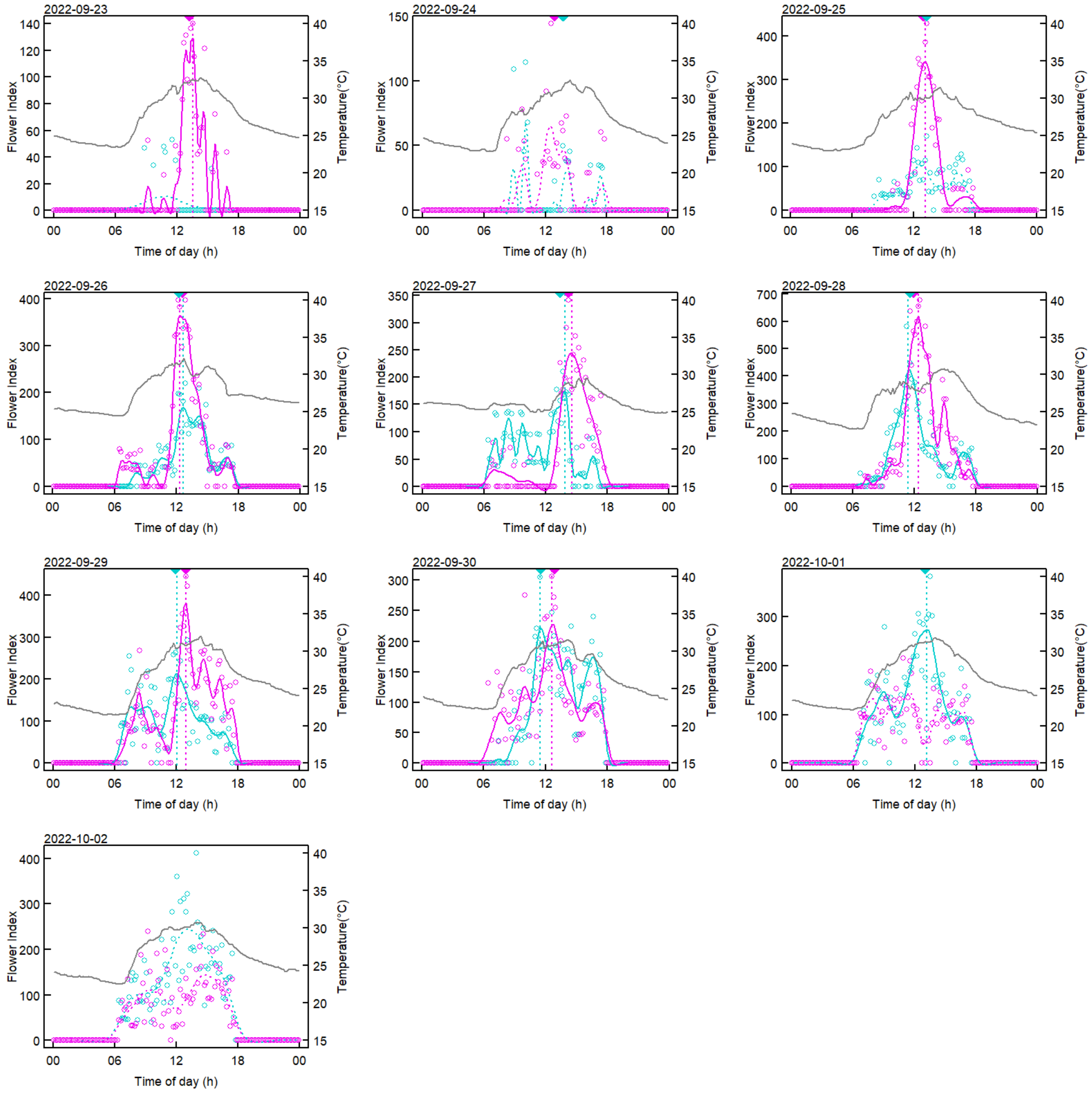

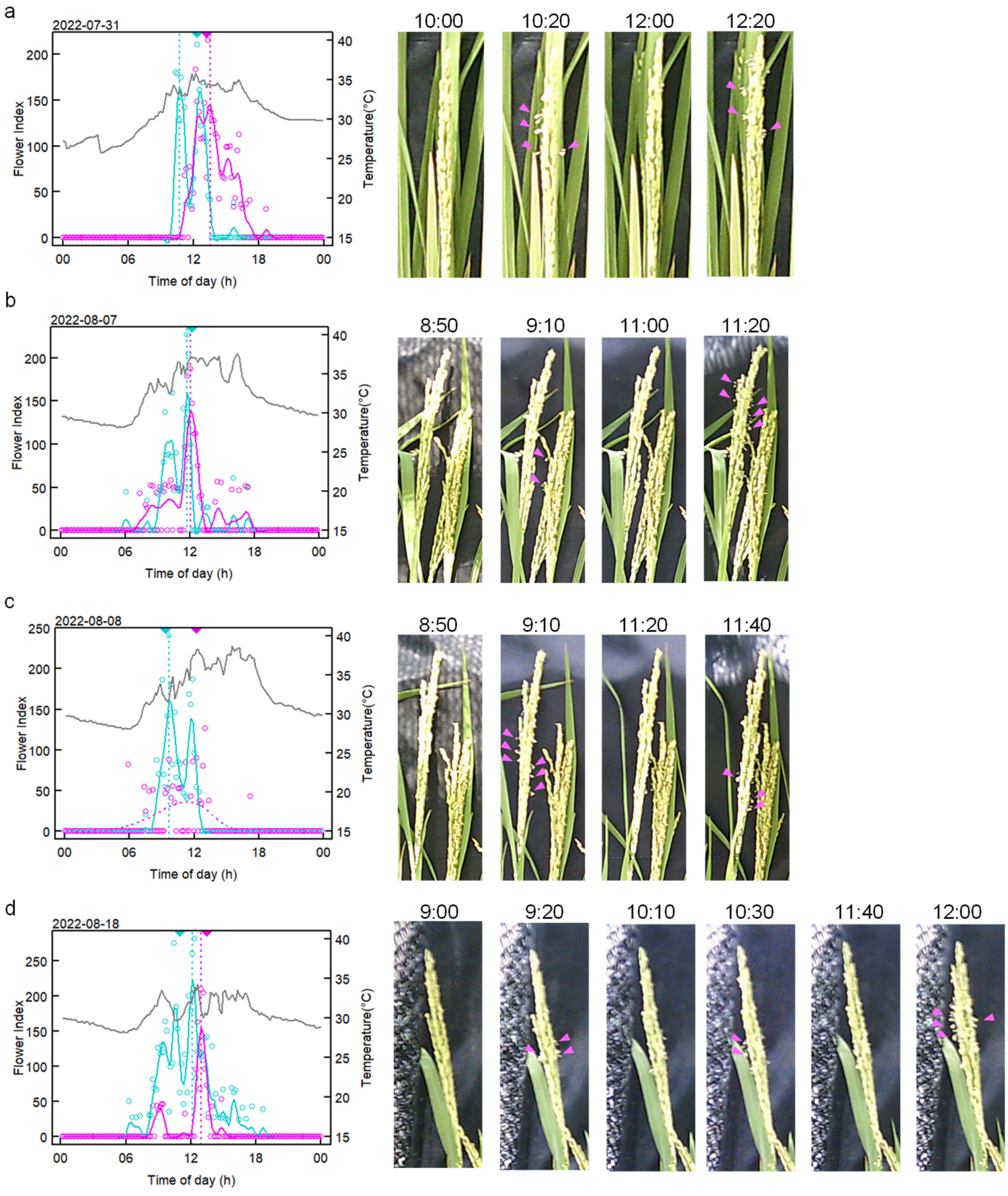

Fig. S2

Diel changes in the flower index and examples of flowering panicles on 2022-7-31 (a), 2022-8-7 (b), 2022-8-8 (c), and 2022-8-18 (d). Cyan and magenta represent IR24 and T65, respectively. Colored lines are smooth splines. Dotted lines and closed triangles represent estimated and observed FOT, respectively. Dotted lines were not shown when the number of detected regions was less than three flowers. Air temperature is shown as a black line. Magenta arrows indicate open flowers.

Table S1.

Sowing and planting dates for plants used in 2022. Plants started heading after approximately two months after planting. The schedules in 2021 were similar to those in 2022.

Year	Cohort	Sowing	Planting	Culture
2022	1	4/10	5/15	Pot
2022	2	4/21	5/22	Pot
2022	3	4/27	5/29	Pot
2022	4	5/4	6/5	Pot
2022	5	5/12	6/13	Pot
2022	6	5/18	6/21	Paddy field
2022	7	5/25	6/28	Paddy field
2022	8	6/1	7/5	Paddy field
2022	9	6/9	7/12	Paddy field
2022	10	6/16	7/12	Paddy field
2022	11	6/22	7/18	Paddy field
2022	12	6/29	7/27	Paddy field

Table S2

Monitoring schedule and layout of plants.

Year	Monitoring start	Monitoring end	Layout
2021	8/13	8/26	T65 T65 IR24 IR24
2021	8/26	9/8	IR24 IR24 T65 T65
2021	9/8	9/21	T65 T65 IR24 IR24
2021	9/21	10/1	T65 T65 IR24 IR24
2021	10/5	10/15	T65 T65 IR24 IR24
2022	7/30	8/12	IR24 IR24 T65 T65
2022	8/12	8/22	IR24 IR24 T65 T65
2022	8/22	9/3	IR24 IR24 T65 T65
2022	9/3	9/14	T65 IR24 IR24 IR24
2022	9/14	9/22	IR24 IR24 T65 T65
2022	9/22	10/5	IR24 IR24 T65 T65
